# The role of the gut microbiota in gastric cancer: the immunoregulation and immunotherapy

**DOI:** 10.3389/fimmu.2023.1183331

**Published:** 2023-06-30

**Authors:** Meiqi Wang, Ge Yang, Yuan Tian, Qihe Zhang, Zhuo Liu, Ying Xin

**Affiliations:** ^1^Department of Gastrointestinal Colorectal and Anal Surgery, China-Japan Union Hospital of Jilin University, Changchun, Jilin, China; ^2^College of Basic Medical Sciences and Key Laboratory of Pathobiology, Ministry of Education, Jilin University, Changchun, China

**Keywords:** gastric cancer, gut microbiota, microbial metabolites, immunotherapy, immune regulation

## Abstract

Gastric cancer (GC) is one of the most common cancers, leading to the deaths of millions of people worldwide. Therefore, early detection and effective therapeutic strategies are of great value for decreasing the occurrence of advanced GC. The human microbiota is involved not only in the maintenance of physiological conditions, but also in human diseases such as obesity, diabetes, allergic and atopic diseases, and cancer. Currently, the composition of the bacteria in the host, their functions, and their influence on disease progression and treatment are being discussed. Previous studies on the gut microbiome have mostly focused on *Helicobacter pylori (Hp)* owing to its significant role in the development of GC. Nevertheless, the enrichment and diversity of other bacteria that can modulate the tumor microenvironment are involved in the progression of GC and the efficacy of immunotherapy. This review provides systematic insight into the components of the gut microbiota and their application in GC, including the specific bacteria of GC, their immunoregulatory effect, and their diagnostic value. Furthermore, we discuss the relationship between the metabolism of microbes and their potential applications, which may serve as a new approach for the diagnosis and treatment of GC.

## 1 Introduction

**Figure 1:**
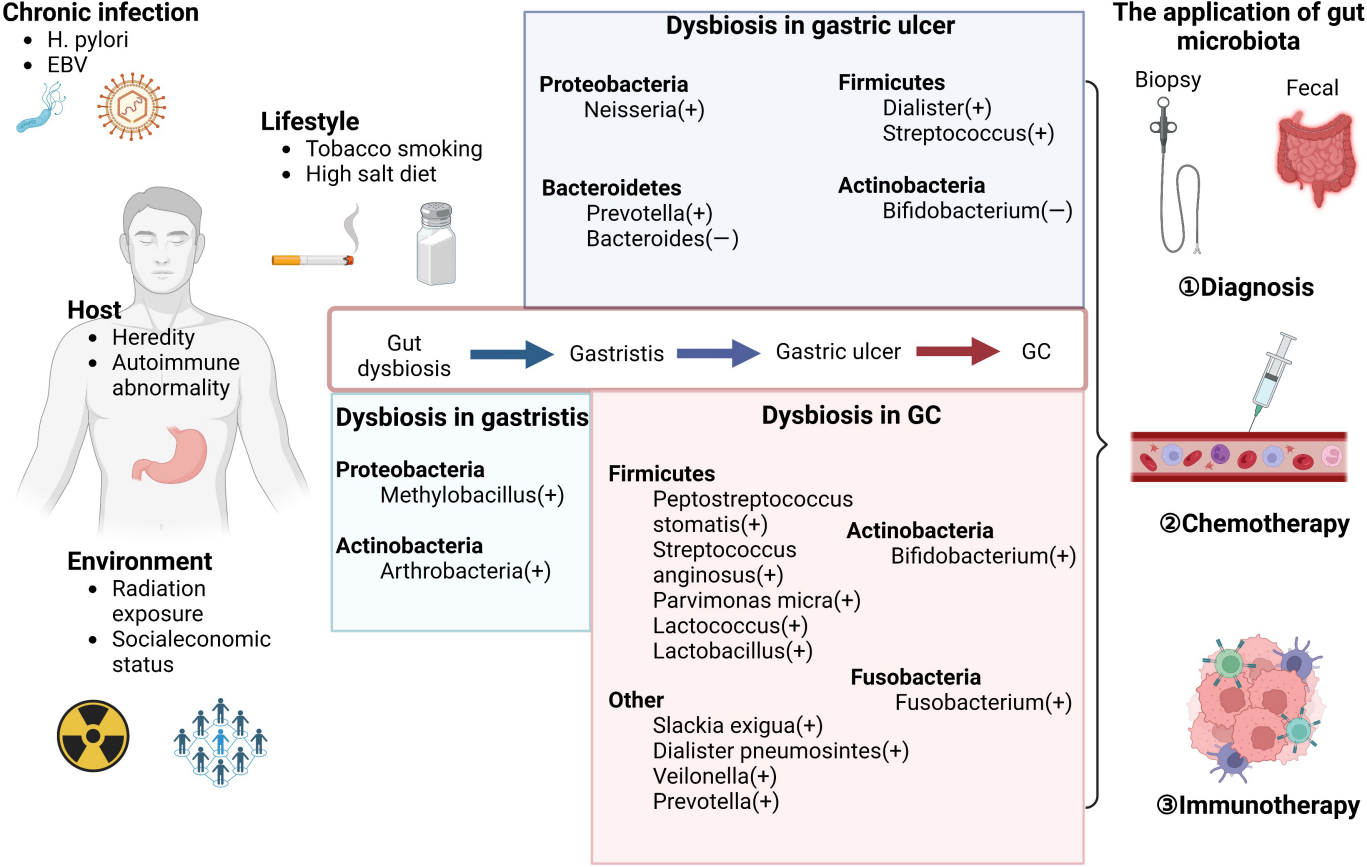
Microbiome dysbiosis in GC. The risk factors can influence the gastric disease progression by shaping the gut microbiota, including chronic infection (such as *H. pylori* infection), lifestyles (such as tobacco smoking and high salt diet), host conditions (such as heredity factors), and environment (such as radiation exposure). The alteration of bacteria except *Hp* in different stages of gastric diseases is shown in the middle of the figure. These evident changes provide novel approaches for early diagnosis in GC. Furthermore, as suggested by existing reports, the abnormal gut microbiota and chemotherapy can have a mutual influence with each other. The gut microbiota also can be used as the biomarkers of the efficacy of immunotherapy to monitor the prognosis in GC.

Gastric cancer (GC) remains the third-most common cause of fatality, with over one million new cases reported annually (1). Infection with *Helicobacter pylori (Hp)* is viewed as the main risk factor for GC, whereas other risk factors include infection with Epstein–Barr virus (EBV), tobacco smoking, a high-salt diet, and heredity (2), which present a complex network of interactions with each other. GC is considered as the result of gastric chronic inflammation and ulcer. Although carcinoembryonic antigen, pepsinogen (PG) 1 and 2, and mucin-like carbohydrate antigens, such as CA199 and CA72.4, can be detected in the serum of patients with GC, they are more useful as prognostic markers than as diagnostic markers because of their low specificity (3). Existing approaches to GC screening are invasive and expensive (4). The treatment of GC mainly includes surgery, chemotherapy, radiation therapy, and molecular targeting, which can improve survival rates. Although treatment regimens for primary tumors and metastases of GC are improving swiftly (5), early detection still plays an important role in GC treatment, making it necessary to explore novel approaches. In recent years, as the complexity and resolution of the human microbiota have improved, more attention has been paid to their functions in tumorigenesis. Commensal microbiota are involved in carcinogenesis and tumor metastasis mainly by modulating metabolism and immune responses (6–9). These mechanisms link host heredity to the environment to facilitate inflammation and mediate immune responses. In this review, we summarize studies on the microbiota of GC to shed light on potential strategies for precise and convenient diagnosis and therapeutic opportunities for GC (**Figure 1**). Given that *Hp* and other gut microbes compromise the efficacy of cancer therapies by modulating the status of immune cells and their secreted inflammatory factors, the gut microbiota may be a new predictive target for GC chemotherapy and immunotherapy (10, 11). This evidence may explain why the outcomes of chemotherapy and immunotherapy are distinct by clarifying the relationship between the microbiota, their metabolism, and GC.

## 2 Alterations of the gut microbiota in GC

In recent decades, the relationship between the gut microbiota community and cancers has been gradually expounded, which has led to the current exploration of the molecular mechanisms of the microbiome in cancer and its application value (12). Furthermore, Manzoor et al. elucidated three layers of relationship between the microbiome and cancer, namely primary, secondary, and tertiary relationships, which are classified based on the distance between tumors and relevant bacteria (13). Most studies have concentrated on colorectal cancer (CRC) because both the species and abundance of intestinal microbiota are high in the colon and rectum compared to other parts of the host. Over the past decade, an increasing number of studies have reported that intestinal microbes affect the development of GC by regulating metabolism and immune signals. Recently, an increasing number of studies have shown that with the development of metagenomics, the potential role of enriched bacteria other than *Hp* in GC has received attention from researchers, which indicates potential application of gut microbiota in GC. The composition of the gut microbiota in GC can be influenced by various factors such as origin, pathological type, phase, and treatment. Therefore, we summarize recent studies on alterations in the microbiota in GC, from both the *Hp* and non-*Hp* perspectives.

### 2.1 Helicobacter pylori in GC

*Hp*, a Gram-negative bacterium, is the major pathogen that causes gastritis, peptic ulcers, GC, and mucosa-associated lymphoid tissue (MALT) lymphoma (14). The prevention and treatment of *Hp* infections pose considerable challenges because they are transmitted *via* the fecal-oral route, and drug-resistant strains have become common. GC can occur in any part of the stomach, including the cardia or non-cardia (fundus, body, or pylorus). There is evidence suggesting that the incidence of non-cardia GC has increased owing to the dysbiosis of gut microbes, especially *Hp*, in addition to epidemiological factors such as age and sex (15, 16). Recently, experts from most countries reached a consensus that eradication of *Hp* should be performed in patients with *Hp* infection to prevent gastric carcinogenesis, preferably before the stage of chronic atrophic gastritis (17–19).

As the major bacterium leading to gastritis, *Hp* can break down urea locally in the gastric mucosa and produce ammonia in an acidic environment. A 40-kb region of chromosomal DNA exists in *Hp*, which encodes a secreted effector protein (CagA) and components of a type IV secretion system, forming its virulence factors (20, 21). During infection with *Hp*, various cytokines present important roles in regulating inflammatory responses. The virulence factors of *Hp* and the secreted cytokines can activate the RAS, MEK, and ERK signaling pathways to cause inflammation and destroy the gastric epithelium, which is a driver of gastric carcinogenesis. However, approximately 1% of the *Hp* infected patients suffer from GC (22). In addition, *Hp* can influence many hormones such as gastrin, leptin, and ghrelin, causing other diseases such as obesity (23).

Microbiota alterations have been identified such that increased microbial richness, decreased microbial diversity, and decreased *Hp* abundance in GC compared to chronic gastritis (24–27). Therefore, *Hp* is considered to prefer healthy gastric mucosa and presents a decreasing trend from gastritis or stomach ulcers to GC (28). Similarly, a vast number of studies examining the microbiota in patients with GC have shown that *Hp* colonization can be found in most GC tissues, which is consistent with previous findings, and Proteobacteria were the second-most common taxon in *Hp*-positive GC tissues (27). *Hp* attributes also have a great impact on the variation in other flora. One study in China observed alterations in the microbiota before and after the eradication of *Hp*, suggesting that Bacteroidetes, Fusobacteria, Actinobacteria, and Firmicutes, which were also the main phyla under normal conditions, showed vital changes (29). Using a series of murine models, researchers validate that infection with *Hp* increased the abundance of Lactobacillales and a variation of myeloid differentiation primary response gene 88 (MyD88, Myd88−/−), causing fast-progressing GC (30). However, non-*Hp* bacteria in GC may also affect *Hp*. A recent study in Japan showed that patients infected with non-*Hp Helicobacter*, whose thallus are longer than *Hp*, were almost negative for *Hp* (31). These direct or indirect changes in the microbiota may participate in the carcinogenesis of the gastrointestinal tract.

**Figure 2:**
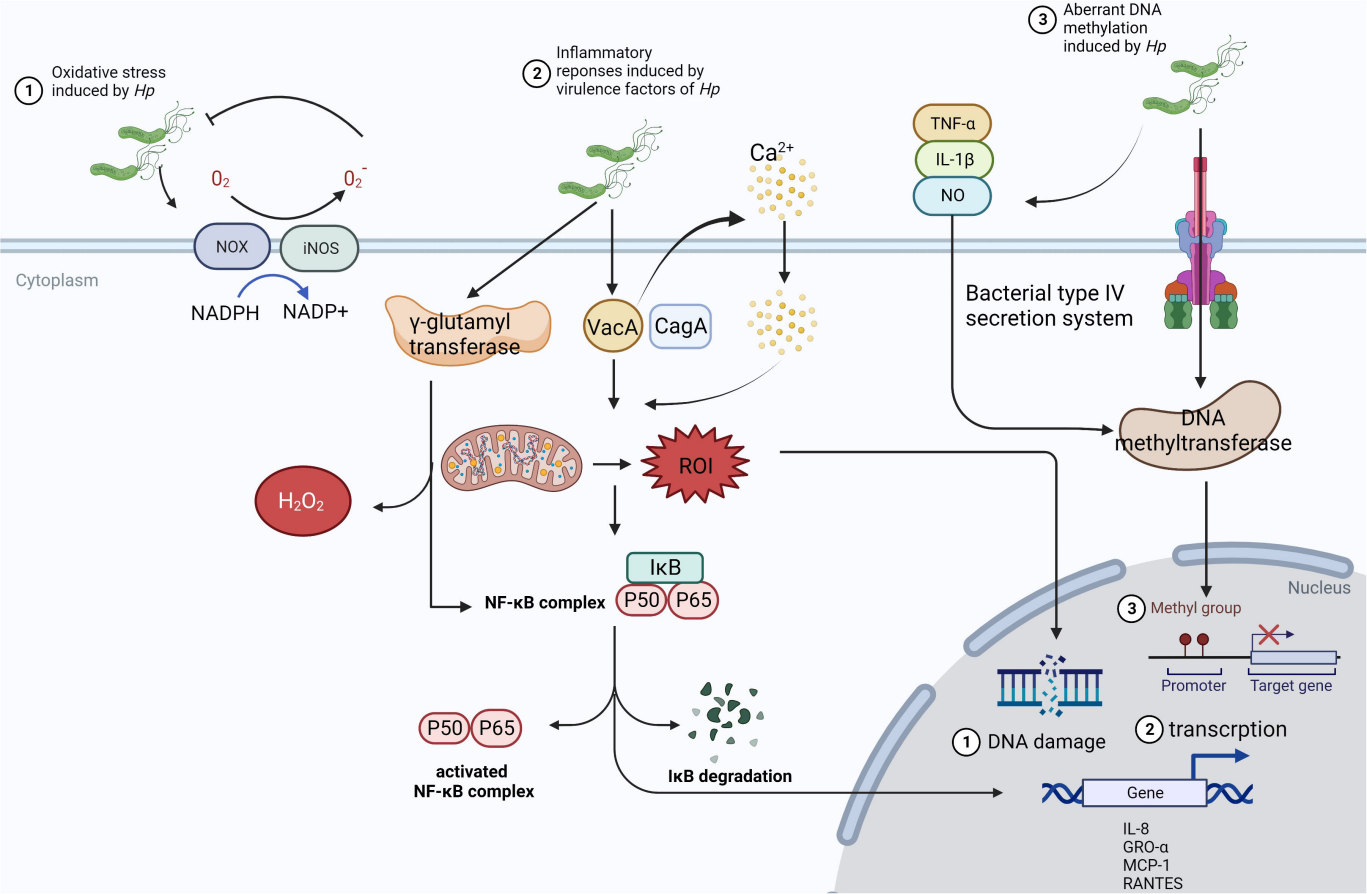
The mechanism of *Hp* infection causing carcinogenesis. ①In the presence of *Hp*, NOX and iNOS are activated to receive the electron from NAPDH, which can be passed to O_2_ to manufacture O_2_^-^ and H_2_O_2_. Then, ROS and RNS could inhibit the growth of microbes and induce. However, this way cannot limit Hp consistently and will cause chronic inflammatory status and tumorigenesis through damaging the gastric mucosa. ②VacA from *Hp* can stimulate Ca^2+^ influx, which promotes the generation of mitochondrial ROI, activates NF-κB signaling, and increases the expression of chemokine to form the infiltration of inflammatory cells. ③*Hp* colonizes in the gastric epithelium, triggering the release of inflammatory cytokines such as TNF-α, IL-1β, and NO. Meanwhile, DNA methyltransferase can be activated in epithelial cells *via* bacterial type IV secretion system, which leads to aberrant hypermethylation of the promoter CpG islands in tumor suppressor genes.

In mechanism, infection of the microbes, especially *Hp*, causing carcinogenesis might be due to inflammatory response, activation of signaling pathways, and alteration of gene expression (**Figure 2**). *Hp* can induce the activation of NADPH oxidase (NOX) and inducible nitric oxide synthase (iNOS) on epithelial cells and neutrophils, leading to the production of reactive oxygen species (ROS) and reactive nitrogen species (RNS), which will kill the bacteria in the lumen (32). Additionally, virulence factors CagA and vacuolating cytotoxin (VacA) of *Hp* could also create oxidative stress microenvironment by the generation of mitochondrial reactive oxygen intermediates (ROI) dependent on Ca^2+^ influx (33). Also as a virulence factor, γ-glutamyl transferase can activate the NF-κB signaling and generate H_2_O_2_ resulting in DNA damage (34). It has been reported that the activity of DNA methyltransferase in epithelial cells can be upregulated and induces aberrant hypermethylation of promoter CpG islands in cancer inhibitor genes to promote carcinogenesis epigenetically (35). Oxidate stress and epigenetic alterations mentioned above have significant association with carcinogenesis induced by *Hp*.

### 2.2 Non-*Hp* microbiota in GC

Growing evidence demonstrates that in addition to *Hp*, other gut microbiota promote carcinogenesis. Although there are a number of studies on the GC microbiota conducted in different countries, the standards of grouping, regional variation, and origin of samples are not unified; therefore, we attempt to summarize recent studies to provide a systematic view on GC microbiota besides *Hp*. The main types of gastric microbiota include Firmicutes, Bacteroidetes, Actinobacteria, and Proteobacteria (36, 37). Under normal conditions, the species of bacteria have a variant in the gastric juice and epithelium owing to the transfer of bacteria from upper to lower anatomical sites. Similarly, the microbiota alterations detected in GC are different because of the different sample sources, including GC tissues, stool, and gastric content. These changes in the major bacteria in the GC contribute to the shape of the tumor microenvironment (TME).

The composition of the microbiota in GC varies with GC subtype. For example, the phyla Fusobacteria, Bacteroidetes, and Patescibacteria were increased in signet-ring cell carcinoma, whereas Proteobacteria and Acidobacteria are mainly identified in adenocarcinoma (38, 39). Histologically, GC can be divided into intestinal and diffuse types, corresponding to differentiated and undifferentiated types respectively. A predominance of *Fusobacterium nucleatum* was found in diffuse type of GC, which was associated with poor survival (40). Although Fusobacteria and Bacteroidetes have been demonstrated to be enriched in cancers, no studies report why these bacteria are increased and how they work. In a study conducted in China, the main pathogens causing gastritis were *Methylobacillus* and *Arthrobacter*, which have co-excluding and co-occurring functions with *Hp*, respectively (25). In addition, researchers have found that in the process of gastric carcinogenesis, dysbiosis occurs among superficial gastritis (SG), atrophic gastritis, intestinal metaplasia, and GC, illustrating the phylum Fusobacteria as the major bacterial composition of GC, which lays the basis for the microbial diagnosis of GC (25, 41–43). This outcome suggests that microbiota are a group of dynamic indices that are not invariable.

The dominant bacteria differ slightly according to the different GC sample origins, such as tumor tissues and fecal samples (44–46). It was demonstrated that between washed and unwashed gastric biopsies, bacterial communities were not significantly different, and non-*Hp* bacterial contamination was transient (47). A study involving 32 patients with SG and 18 patients with GC suggested that the microbiota of patients with GC are different from those of patients with SG, showing an increased abundance of *Dialister* spp., *Helicobacter* spp., and *Lactobacillus* spp. as well as a decreased abundance of *Fusobacterium* spp. at the genus level, whereas the change in Fusobacteria showed opposite outcomes according to two different methods of sampling: gastric wash samples and biopsy samples (41, 48). However, there was no difference between GC tissues and para-cancerous sites, indicating that changes in bacteria occur at the early stage of tumorigenesis rather than during the progression from the early to late stages of the tumor (48, 49). These studies revealed that enrichment of specific bacteria might contribute to GC initiation and progression through changes in metabolic patterns, such as increased nucleotide metabolism and nitrogen-containing compounds (50, 51). Using wild-type germ-free mouse models, Kwon et al. found that intestinal metaplasia microbiome transplantation induced GC by augmenting F4/80, Ki-67, and CD44v9/GSII lectin expression (44). This mechanism may be explained by the activation of inflammasomes by bacteria. The composition of bacteria such as T3SSs and flagellin could be recognized by inflammasomes, leading to the release of inflammatory factors, such as IL-1β, followed by the movement of bacteria or the death of infected cells (51). Furthermore, a study revealed that fecal *Desulfovibrio* in patients with GC produced H_2_S, contributing to the release of inflammatory factors that promote carcinogenesis (46). Sensor histidine kinases, which can be increased by bacterial defense to help the microbiota adapt to the TME, increase during GC progression (52).

Additionally, the enrichment of cancer microbiota and dominant flora are different owing to different human single nucleotide polymorphisms among people from different regions; for instance, in Asia, Firmicutes was the main phylum, whereas in Europe, it was Proteobacteria, and both in Europe and East Asia, the genus *Bacillus* was the dominant flora (53). In addition to the pathological types and hereditary factors, GC treatment can alter the microbiota community structure of feces, which is involved in postoperative comorbidities and prognosis. It has been firstly studied through metabolomics and metagenomics that patients after gastrectomy have a high level of aerobes, facultative anaerobes (*Escherichia*, *Enterobacter*, and *Streptococcus*), and oral microbes (*Streptococcus* and *Enterococcus*) in the fecal samples (45, 54). The regulatory mechanism of altered non-*Hp* levels after surgery is associated with the metabolism of nucleotides and amino acids, nitrification, adoption of the TME, inflammation, and immune modulation (45). For instance, after gastrectomy, the activity of phosphate and several amino acids, such as lysine, arginine, and ornithine, is upregulated, accompanied by the enrichment of *Atopobium*, *Veillonella*, and *Streptococcus* (45). Therefore, based on these sequencing outcomes of samples from patients with GC, we found that the phyla Proteobacteria and genera *Lactococcus*, and *Prevotella* were enriched in GC biopsies (**Table 1**), whereas the genus *Helicobacter* was decreased in GC biopsies, which was consistent with the fact that the TME in GC tended to develop in a direction suitable for non-*Hp* growth. The infection of *Hp* could lead to dysbiosis. A previous study has demonstrated that positive *Hp* status is associated with the enrichment of *Proteobacteria, Spirochetes, Acidobacteria* and the decrease of *Actinobacteria, Bacteroidetes, Firmicutes* (57). *Akkermansia* is also found in the caecal and colonic lumen after long-term *Hp* infection, which leads to the degradation of mucus (58). However, the interaction between *Hp* and non-*Hp* bacteria and the mechanism of alteration tendencies remain unclear (59). Here, a synthetic analysis of the GC microbiota, mainly including the upregulated and downregulated microbes and their correlated mechanisms, is presented to provide a general understanding (**Table 2**).

**Table 1:** The components of the gut microbiota and their application in GC.

Phylum level	Genus level	Application	References
**Firmicutes**	*Lactococcus*	lactic acid production as an energy source for tumorigenesis	(26)
	*Lactobacillus*		(26, 55)
	*Lachnospiraceae*	–	(55)
	*Peptostreptococcus*	Biomarkers for GC diagnosis	(25)
	*Dialister*		
	*Mogibacterium*		
**Proteobacteria**	*Escherichia–Shigella*	–	(55)
	*Burkholderia fungorum*	–	(55)
	*Moryella*	Prediction of the progression of EGN	(52)
	*Halomonas*	Biomarkers for identifying GC subtypes	(38)
	*Shewanella*		
	*Betaproteobacteria*	To develop strategies for prevention, early diagnosis and treatment of GC	(35)
	*Gammaproteobacteria*		
**Bacteroidetes**	*Prevotella 7*	A new biomarker and/or preventive target for GC	(56)
**Nitrospirae**	*Nitrospira*	Increasing the function of nitrate reductase and nitrite reductase to facilitate gastric malignant transformation	(38, 55)

– means that no applications are reported except major gut microbiota.

**Table 2:** Gut microbiota dysbiosis in GC.

Sample Size	Country and District	Origin of Sample	Sequencing Methods	Decreased bacteria Composition in GC	Increased Non-Helicobacter bacteria Composition in GC	Correlated mechanisms	References
**12 GC patients and 20 functional** **dyspepsia**	Singapore and Malaysia	Gastric antral biopsies	16S rRNA gene amplicon sequencing	*Methylobacterium*	*Lactococcus, Veilonella*, *Fusobacterium*	Elevated level of lactate Increased carbohydrate digestion and absorption	(26)
**160 GC patients**	China	Gastric tumor tissues and matched non-malignant tissues	16S rRNA gene amplicon sequencing	–	*Helicobacter, Proteobacteria*	Membrane transport of metabolites	(27)
	Mexico				*Helicobacter, Proteobacteria* and *Firmicutes*		
**81 chronic gastritis, 54 GC patients**	Portuguese	Gastric biopsies and non-malignant tissues adjacent to the tumor	16S rRNA gene profiling	*Helicobacter* and *Neisseria*	*Phyllobacterium* and *Achromo-* *Bacter*, *Xanthomonadaceae*, *Enterobacte-Riaceae, Lactobacillus, Clostridium, Rhodococcus*	Increased nitrification	(24)
**21 SG, 23 AG, 17 IM, 20 GC patients**	Xi’an, China	Gastric biopsies and matched non-malignant tissues	16S rRNA gene amplicon sequencing	*Vogesella*, *Candidatus_Portiera*, *Comamonadaceae*, *Acinetobacter*	*Peptostreptococcus* *stomatis, Streptococcus anginosus, Parvimonas micra*, *Slackia exigua, Dialister pneumosintes*	Enhanced nucleotide metabolism Inflammasomes activation	(25, 44)
**6 GC and 5 SG patients**	China	Gastric wash samples	shotgun metagenomic sequencing	*Sphingomonadaceae*	*Neisseria*, *Alloprevotella*, *Aggregatibacter*	Enhanced LPS biosynthesis	(41)
**276 patients with GC**	China	Gastric tumor tissues and matched normal tissues and peritumoral tissues	16s rRNA gene sequencing	–	*Prevotella melaninogenica*, *Streptococcus anginosus*, *Propionibacterium acnes*	Increased transportation and metabolism of nucleotide and amino acid	(49)
**62 GC patients**	China	Gastric tumor tissues and matched non-malignant tissues	16s rRNA gene sequencing	–	Genus *Streptococcus*, *Peptostreptococcus*, *Prevotella*, *Prevotella_7 Acinetobacter*, *Bacillus*, *Selenomonas*, *Lachnoanaerobaculum*	Increased energy metabolism and concentration of nitrogen-containing compounds	(50)
**64 GC patients**	China	Gastric tumor tissues and matched normal tissues and peritumoral tissues	bacterial genomic DNA sequencing	*Staphylococcus* and *Corynebacterium*	*Streptococcus*, *Peptostreptococcus*, *Lactobacillus*, *Bifidobacterium*, *Neisseria*, *Veillonella*, *Shewanella*	Changed metabolism patterns, immune system dysfunction	(60)
**57 *Hp* positive patients, 58 *Hp* negative patients after treatment, and 49 *Hp* negative patients**	China	Gastric biopsies	16s rRNA gene sequencing	*Proteobacteria*, *Epsilonproteobacteria*, *Campylobacterales*, *Helicobactera*ceae, *Helicobacter*	*Cyanobacteria/Chloroplast, Bacteroidetes, Fusobacteria, Actinobacteria*, *Firmicutes*	Glycan degradation, carbohydrate and protein digestion and absorption after *Hp* eradication	(29)
		Stool samples		*Bacteroidales*	*Clostridiales* and *Bifidobacterium*		
**120 noncancer** **patients, 48 GC patients**	Mongolia	Gastric biopsies and matched non-malignant tissues	16S rRNA gene amplicon sequencing	–	*Enterococcus*, *Lactobacillus*, *Firmicutes*	Increased activity of the Embden-Meyerhof-Parnas pathway and utilization of sugar	(42)
**22 patients with dyspepsia, 12 GC patients**	Denmark	Gastric antral biopsies	16S rRNA gene-targeted amplicon sequencing	*Actinomyces* spp.	*Streptococcus*	Consistent *Hp* infection	(47)
**30 healthy controls (HC), 21 non-** **atrophic chronic gastritis (CG), 27 IM, 25 IN, and 29** **GC patients**	China	Gastric biopsies and gastric tumor tissues	16s rRNA gene sequencing	Aerobic and facultatively anaerobic bacteria	*Lactobacillus*, *Streptococcus*, *Prevotella*, *Veillonella*	Decreased nitrate/nitrite reductase functions and nitrate accumulation	(61)
**375 tumors and 27 matched normal tissues**	Europe	Gastric cancer samples and around tissues	TCGA and GTEx data analysis	*Helicobacter*	*Bacillus, Parasutterella, Brevibacillus, Fusobacterium, Enterobacter, Cloacibacterium*, *Suterella*	Induction of an autolysosome to inhibit invasive bacteria	(53)
	Asia				*Firmicutes*		
**50 patients with a history of gastrectomy for gastric cancer and 56 control patients**	Tokyo, Japan	Stool samples	shotgun metagenomics sequencing	–	*Streptococcus* spp.*, Prevotella* spp.*, Veillonella* spp., *Lactobacillus* spp.	Abnormal transportation of raffinose/stachyose/melibiose, isoleucine biosynthesis, and cobalamin biosynthesis	(45, 54)
**61 healthy individuals, 83 patients with GC**	Hangzhou, China	Stool samples	16s rRNA gene sequencing	–	*Lactobacillus*, *Megasphaera*	–	(43)
**35 healthy people, 38 patients with GC**	Shandong, China	Stool samples	Fecal samples DNA sequencing	*Faecaliberium*, *Roseburia*, *Lachnospira*, *Anaerostipes*	Genera *Enterobacteriaceae*, *Streptococcaceae*, *Desulfovibrio*	NO, IL-1β and IL-18 production induced by H_2_S	(46)
**32 superficial gastritis (SG) patients, 18 GC patients**	Nanjing, China	Gastric biopsies; gastric tumor tissues and matched non-malignant tissues	16s rRNA gene sequencing	*Fusobacterium* spp.	*Dialister* spp., *Helicobacter* spp., *Lactobacillus* spp.*, Rhodococcus* spp.*, Rudaea* spp., *Sediminibacterium* spp.	Increased nitrate reductase	(48)
**43** **participants**	Singapore	Gastric biopsies	16s rRNA gene sequencing	*Lactobacillus* and *Bifidobacteria*	*Phyllobacteriaceae*, *Enhydrobacter*, *Moryella*	Reduced galactose, sucrose and starch metabolism	(52)
**37 patients with GC**	China	Gastric tumor tissues and matched normal tissues	16s rRNA gene sequencing	*Helicobacter*	*Lactobacillus, Streptococcus, Acinetobacter, Prevotella, Sphingomonas, Bacteroides*, *Fusobacterium*	Increased metabolites such as amino acids, carbohydrates and carbohydrate conjugates, fatty acyls, glycerophospholipids, nucleosides, and nucleotides	(62)
**13 proximal and16 distal GC patients**	China	Gastric tumor tissues and matched normal tissues	16S rRNA amplicon sequencing	In proximal GC: *Rikenellaceae_RC_gut_group* In distal GC: *Methylobacterium-Methylorubrum*	In proximal GC: *Helicobacter* In distal GC: *Helicobacter*	In proximal GC: hormone metabolism In distal GC: the sphingolipid signaling pathway, arginine biosynthesis and etc.	(63)
**5 patients each of non-atrophic gastritis (NAG), IM and intestinal-type GC**	Mexicano	Gastric biopsies	16S rRNA microarray	TM7, *Porphyromonas* and *Neisseria*	*Lactobacillus coleohominis* and *Lachnospiraceae*	–	(64)
**5 dyspeptic control patients and 10 GC patients**	Sweden	Gastric biopsies	T-RLFP, 16S rRNA gene sequencing	–	*Streptococcus*	Formation of N-nitroso compounds	(65)

– means that decreased bacteria and correlated mechanisms are are studied.

## 3 The effect of gut microbial metabolite on GC

Similar to gut microbes, their metabolites can modulate inflammation and even carcinogenesis *via* different mechanisms, such as disrupting the balance of anti-inflammatory and pro-inflammatory signaling pathways and forming a functional complex of biomolecules in carcinogenesis (66). Nevertheless, metabolites of commensal bacteria can promote or inhibit tumor progression (67). For instance, *Clostridium scindens* in the digestive tract can break down not only fat into secondary bile acid for carcinogenesis, but also fiber into butyrate for antitumor effects. In addition, other metabolites produced by gut bacteria can provide diagnostic and prognostic guidance. For example, in addition to the common phyla Proteobacteria and Fusobacteria, polyamines were also identified to be enriched in CRC, which sheds light on their diagnostic significance in CRC (68). p53 is a common tumor suppressor gene, the mutation of which leads to tumorigenesis. Nevertheless, it promotes carcinogenesis in the proximal gut and tumor organoids (69). Gallic acid, a polyamine produced by *Lactobacillus plantarum* and *Bacillus subtilis*, is necessary for the tumor-suppressive function of mutant p53, providing new ways to prevent oncogenesis *via* diet management (69).

Some studies have focused on the effects of microbial metabolites on GC development, finding that some common metabolites, such as galactose and amino acids, are enriched in patients with GC, followed by changes in bacteria, which are involved in the carcinogenesis and progression of GC (**Table 3**). After identifying the specific bacteria involved in the development of GC, the metabolic pathways of the microbiota were identified, and the molecular mechanisms were explored further. Lactic acid bacteria (LAB) are a group of bacteria widely distributed in nature. LAB can produce large amounts of lactate from fermentable carbohydrates, which is essential for physiological functions and tumorigenesis. *Lactobacillus*, a genus of LAB, is determined as the dominant group of bacteria in GC (42), and its species are involved in the progression of GC *via* lactate production, bile secretion, and amino acid metabolism pathways (62). Furthermore, lactobacilli in GC can increase the production of exogenous lactate and reactive oxygen species to provide tumor cells with energy and facilitate epithelial-mesenchymal transition, facilitating GC cell growth and metastasis (70). This evidence suggests that lactate is a significant metabolite affecting GC. However, Hu et al. showed that short chain fatty acids (SCFAs) and branched amino acids are involved in the development of SG, but not GC (41). Butyrate, an SCFA, is a common metabolite associated with gut microbiota activity. It has been demonstrated that butyrate produced by gut microbiota can induce CRC (71) *via* the fermentation function (72). However, another SCFA, sodium acetate, induces CRC and gastric adenocarcinoma cell apoptosis, depending on the Fas receptor (FasR)/Fas ligand (FasL) (73). The outcomes of SCFAs and other microbial metabolite functions in cancer may require a larger sample size for verification.

**Table 3:** Discriminative metabolites detected in patients with GC.

Country	Samples	Screening technique	Discriminative metabolites	Outcomes	References
Mongolia	48 GC and 120 noncancer patients	KEGG pathways analysis	Lactate↑ N-nitroso compounds↑	Progression of GC	(70)
China	30 healthy controls (HC), 21 non- atrophic chronic gastritis (CG), 27 IM, 25 IN, and 29 GC patients	KEGG pathways analysis	Nitrate and nitrite↑	Carcinogenesis of GC	(61)
Singapore	IM and early gastric neoplasia (EGN) patients	16S rDNA	Galactosamine PTS system EIIB component (agaB, agaC, agaD)↓ Arginine↑	Carcinogenesis and progression of GC	(52)
China	37 paired GC tissue samples	UHPLC-MS/MS	1-methylnicotinamide and N-acetyl-D-glucosamine-6-phosphate↑ Amino acids↑ Carbohydrates and carbohydrate conjugates↑ Glycerophospholipids↑ Adenosine↑	Development and progression of GC	(62)
China	Gastric wash samples from 6 GC and 5 SG patients	Shotgun metagenomic sequencing	Lipopolysaccharide (LPS)↑ L-arginine↑	Carcinogenesis of GC	(41)

↑ means the metabolites are increased and ↓ means decreased.

In addition to these fatty acyl class metabolites, amino acids, carbohydrates and their conjugates, fatty acids, and glycerophospholipids also play significant roles in the progression of GC. Dai et al. demonstrated that 150 metabolites showed significant differences and were more abundant in GC, supporting the hypothesis that metabolites play important roles in carcinogenesis (62). From non-atrophic chronic gastritis to GC, the function of nitrate reductase is weakened, which is associated with changes in the dominant gut microbes in the gastric mucosa along with the progression of GC (61). Whether these metabolites increase or decrease the risk of GC and the regulatory mechanisms of microbial metabolites require further exploration.

## 4 The immunoregulatory effect of the gut microbiota and their metabolites on GC

### 4.1 The immunoregulatory effect of the gut microbiota on GC

**Figure 3:**
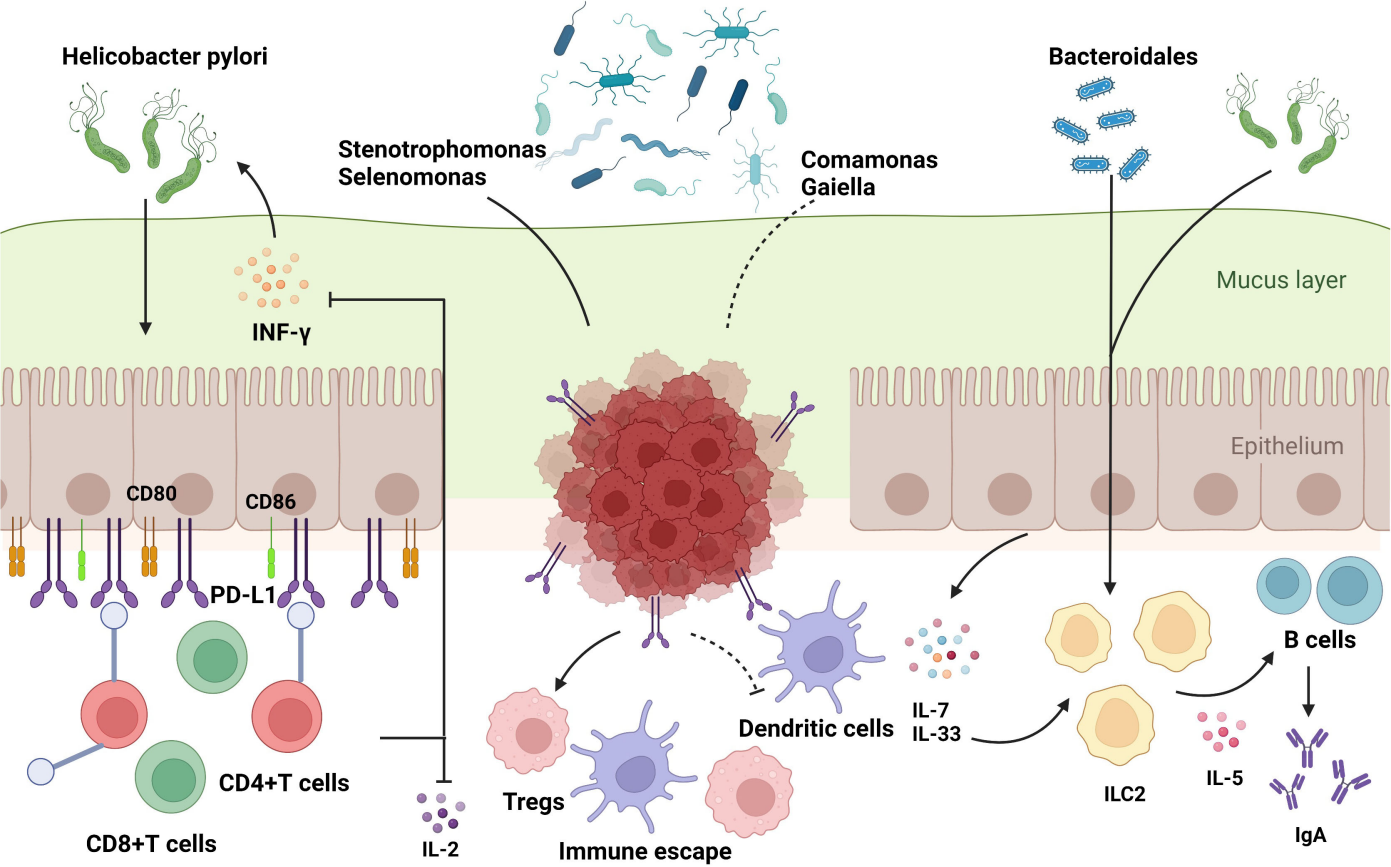
The influence of gut microbiome on immune regulation in stomach. The commensal bacteria are important for maintaining immune homeostasis of the intestine. Gut microbiota could modulate the innate immunity and the adaptive immunity to influence TME, which activates or suppresses immune responses to have an impact on GC development. Some specific microbiota supports the growth of gastric immunosuppressive cells such as BDCA2^+^pDCs and Foxp3^+^Tregs to induce the occurrence of immune escape. The bacteria could enter the mucus layer of the stomach to influence the functions of adaptive immune cells by regulating the associated molecules, which include: 1) upregulation of immune-related receptors such as CD80, CD86, and PD-L1 on gastric epithelial cells; 2) increased manufacture of regulatory factors such as IL-7 and IL-33. Based on these molecular mechanisms, gut microbiota could exert an impact on T and B cells to support the bacteria or to stimulate the protective response in host, which play a significant role in carcinogenesis and immunotherapy responses in GC.

It is already known that both dysbiosis of gut microbes and dysfunction of the immune system, including innate and adaptive immune responses, can result in cancer; however, the relationship between the microbiota and the immune system remains unclear. It is generally believed that the dysbiosis of gut microbiota could break the balance of gut immune environment, leading to inflammation and carcinogenesis. Differences among microbiota are dependent on the pattern recognition receptors (PRRs) located on innate immune cells, which distinguish favorable bacteria from harmful ones by recognizing pathogen-associated molecular patterns (PAMPs), which are bacterial endotoxins or lipopolysaccharide of bacteria (36). Gut microbiota can be transported by various cells in the gut lumen to combine with specific PRRs to initiate T or B cell responses (6, 74). *Hp* infection can upregulate the expression of CD80 and CD86 in gastric epithelial cells, activating T-cell responses (75). Furthermore, it has been reported previously that *Hp* inhibits the proliferation of CD4+T cells and reduces the synthesis of IL-2 and IFN-γ by upregulating the expression of programmed cell death-ligand 1 (PD-L1) on gastric epithelial cells (76, 77), which might explain the effect of immunotherapy, especially immune checkpoint inhibitors (ICIs), by influencing the activity of immune cells such as dendritic cells and macrophages (10). Moreover, CD8+ tissue-resident memory T cells (TRM) in the TME were suppressed by *Methylobacterium* in GC tissues, accompanied by the limitation of TGF-β expression (78). In addition to the T cell response, *Hp* infection can induce the production of IgA by B cells by activating group 2 innate lymphoid cells (ILC2s) (79). A retrospective study demonstrated that *Stenotrophomonas* and *Selenomonas* are positively correlated with BDCA2+ plasmacytoid dendritic cells (pDCs) and Foxp3+ Regulatory T cells (Tregs), whereas *Comamonas* is negatively correlated with BDCA2+ pDCs, which are involved in the immune escape of GC cells (60). Taken together, these results provide a theoretical framework for identifying the correlation between the gut microbiota and immunoregulation in GC (**Figure 3**).

### 4.2 The immunoregulatory effect of gut microbial metabolites on GC

Not only can the gut microbiota modulate immune responses during tumorigenesis, but their metabolites also play a significant role in cancer progression and immunity. Legoux et al. found that the metabolite 5-(2-oxopropylideneamino)-6-d-ribitylaminouracil promoted the growth of mucosal-associated invariant T (MAIT) cells through transmission from mucosal surfaces to the thymus, suggesting a positive role in enhancing the protective immune response of the body (80). The effect of gut microbial metabolites on tumors modulates immune cell phenotypes and functions by regulating immunosuppressive cytokine secretion (81). These metabolites can inhibit the expression of histone deacetylase and strengthen immune cell functions by binding to their receptors, such as GPR41 and GPR43, on immune cells, especially effector T cells (67, 82, 83). Yang et al. demonstrated that SCFAs facilitate IL-22 production in CD4+ T cells by binding to GPR41 and inhibiting histone deacetylase, which could inhibit the inflammatory response (84). SCFAs also maintain gut homeostasis by facilitating the production IL-10 by Th1 cells (82, 85, 86). In addition, hydroxycitrate produced under starvation conditions can improve the efficacy of mitoxantrone in a T cell-dependent manner (87). Similarly, a study found that Roux-en-Y reconstruction for radical gastrectomy in patients with GC elevated the level of butyrate to suppress the activation of macrophages by downregulating the inflammasome NLRP3 and inhibiting the secretion of pro-inflammatory mediators (88), suggesting that SCFAs play an important role in the prevention of colitis after GC surgery.

## 5 Diagnostic and therapeutic options for targeting the gut microbiota on GC, especially immunotherapy

Increasing evidence suggests that gut microbiota plays a significant role in GC therapy. The role of the microbiota and their metabolites in modulating the efficacy of anticancer treatment has been elucidated, and the gut microbiota not only impede harmful bacterial invasion but also influence the efficacy of chemotherapy and immunotherapy.

### 5.1 The role of the gut microbiota on immunotherapy of GC

Gut microbiota can affect the efficacy of tumor immunotherapy, including immune checkpoint blockade (ICB) therapy, allogeneic hematopoietic stem cell transplantation (allo-HSCT) and adoptive cell transfer (ACT) methods. Although ICIs shows great promise in the treatment of refractory cancers including GC, the resistance after ICB therapy limits its broad application (89). Choi et al. found that ICB therapy induced the enhanced anti-tumor immune responses by translocating into tumor and secondary lymphoid organs of gut bacteria such as *Bifidobacterium*, *Streptococcus*, and *Lactobacillus* spp (90). Taking CTLA-4 blockade for example, B. fragilis can produce zwitterionic poly-saccharides dependent on interleukin-12 to promote T helper 1 (TH1) immune responses (91). Patients treated with allo-HSCT including irradiation, chemotherapy and immunosuppression therapy present the decreased diversity of gut microbiota due to the release of inflammatory cytokines, damage-associated molecular patterns (DAMPs) and pathogen-associated molecular patterns (PAMPs), which can be viewed as the predictor of mortality (92, 93). Furthermore, the efficacy of ACT can be enhanced by the gut microbiota and their metabolites such as SCFAs, which suppresses class I histone deacetylase epigenetically and promotes the activity of mTOR complex metabolically of CD8+ T cells (94).

With the discovery of immunotherapeutic targets for GC, many drugs targeting Human Epidermal Growth Factor Receptor 2 (HER2), Vascular Endothelial Growth Factor Receptor 2 (VEGFR2), cytotoxic T-lymphocyte-associated antigen 4 (CTLA-4), PD-1, and programmed cell death ligand 1 (PD-L1) are already available in the market or in clinical trials. Microbes can influence the metabolism of drugs through chemical modifications (95) and bioaccumulation (96). Some studies have confirmed that the microbiota and their metabolites may have a broad impact on anti-GC immunotherapy, mediated by the secretion of cytokines and enhancement of T cell infiltration (97, 98). Therefore, owing to the assistive role of the gut microbiota in anticancer therapy, the efficacy of cancer immunotherapy may be impaired by the use of antibiotics (99), together with changes in the dominant gut microbiota, which helps to enhance the antitumor immune response (100). Researchers, using methods of sequencing fecal samples, differentiated responders from non-responders to ICIs and illustrated that specific microbes (101) might be linked with increased immunity and immune cell infiltration in tumors (98, 102–104). For example, gut microbes have been demonstrated to be enriched in patients who show a strong response to anti-PD-1 therapy and induce increased manufacturing of memory CD8+ T cells and natural killer cells in advanced non-small cell lung cancer (NSCLC) patients in China (105). Regarding the gut microbiota–immune system axis, the response rate to immunotherapy can be influenced by the gut microbiota through numerous mechanisms. GC can be divided into four types: EBV-positive, microsatellite instability (MSI), genomically stable, and chromosomal instability (106). A large-scale microbial profiles of GC from two demographically distinct cohorts has unveiled that *Selenomonas*, *Bacteroids*, and *Porphyromonas* are the top three microbes in MSI-high GC patients (107). A clinical trial attempted to associate molecular characterization with the response rate of immunotherapy to pembrolizumab, a PD-1 inhibitor, and demonstrated that high-level microsatellite instability or EBV positivity is a predictive target (108). Furthermore, in addition to high-level microsatellite instability and positive EBV status, *Hp* infection is not only an indicator of high PD-L1 expression but also of poor prognosis after immunotherapy by suppressing innate and adaptive immune responses (77, 109), which might be used as an index for predicting immunotherapy efficacy in GC patients. However, the mechanisms underlying the regulatory effect of *Hp* on immunotherapy remain unknown because the two studies mentioned above seem to display contradictory results.

With the increasing use of fecal microbial transplantation (FMT) for disease treatment, the important role of microbes in tumor immunotherapy has been confirmed. For instance, in germ-free mice, FMT helps non-responders to PD-L1 immunotherapy increase immune infiltration and improves the efficacy of anti-PD-L1 therapy in melanoma (67). In addition, probiotic intervention is attracting more attention for immunoregulation recently. For example, supplement of *Lactobacillus kefiranofaciens* ZW18 (ZW18) could significantly enhance the effect of PD-1 inhibitor treatment by activating immune responses (110). Furthermore, Kassayová et al. found that *Lactobacillus plantarum* inhibited the proliferation of breast cancer cells by increasing the levels of CD8+ T cells and CD4+ T cells (111). However, Spencer et al. evaluated the influence of the use of probiotics on melanoma patients treated with ICB therapy in preclinical models and found that probiotics *Bifidobacterium longum* might not only no benefit for tumor patients, but also damaging the efficacy of immunotherapy (112). Vétizou et al. reported that *Bacteroides thetaiotaomicron* and *Bacteroides fragilis* guarantee the efficacy of ipilimumab, a monoclonal antibody against CTLA-4, and reduce adverse responses such as colitis in melanoma (85, 91, 113). *B. fragilis*, a well-known opportunistic pathogen, is divided into enterotoxigenic *B. fragilis* (ETBF) and nontoxigenic *B. fragilis* (NTBF), whose difference is based on whether it produces ETBF virulence factor *B. fragilis* toxin (BFT) or not. ETBP can activate NF-κB and Stat3 signaling promoting carcinogenesis, while NTBF can produce Polysaccharide A (PSA) delivered by outer membrane vesicles and internalized by antigen-presenting cells (APCs) to activate Tregs and CD4+ T cells (114, 115). Therefore, PSA from *B. fragilis* might restore the immune responses of anti-CTLA4 therapy.

### 5.2 The potential effect of the gut microbiota on chemotherapy of GC

There is emerging evidence that the microbiota can affect chemotherapy by mediating resistance to drugs such as cyclophosphamide and gemcitabine; conversely, they can be influenced by these drugs (116, 117). Furthermore, the gut microbiota can inhibit the side effects of chemotherapy. Oxaliplatin is a common drug used in chemotherapy for digestive tract tumors and is usually used in combination with 5-fluorouracil and leucovorin. Shen et al. found that oxaliplatin-induced pain can be relieved by suppressing the gut microbiota through the LPS-TLR4 pathway (118). It has been known that lipopolysaccharide (LPS) is immunogenic stimulus of immune cells including B cells, monocytes, macrophages, and other LPS-reactive cells. The regulatory process of the gut microbiota and bacterial components, such as LPS, in cancer chemotherapy may be associated with the activation of anticancer immune responses mediated by immune cells. In turn, 5-fluorouracil contributes to intestinal mucositis by decreasing the richness and diversity of the gut microbiota, with a lower abundance of Firmicutes and Proteobacteria (119). As mentioned above, gut microbial metabolites in GC, such as SCFAs, activate immune responses and regulate host immunity. In conclusion, the gut microbiota and their metabolites may affect the efficacy of chemotherapy in GC.

## 6 Potential of the gut microbiota as biomarkers of GC

Advanced sequencing methods for the gut microbiota represent an opportunity for early GC diagnosis. It has been reported that the genera *Desulfovibrio, Escherichia*, *Faecalibacterium*, and *Oscillospira* in feces present the main variation in GC, which has the potential for GC diagnosis (46). Another example of a microbial diagnostic biomarker is the presence of the two genera *Lactobacillus* and *Megasphaera*, which are enriched in patients with GC and have diagnostic value in distinguishing patients with GC from healthy individuals (43). In a multicenter, large-sample observational study, *Streptococcus anginosus* and S*treptococcus constellatus* were verified as early warning biomarkers for GC (120). In addition to identifying potential microbial biomarkers of GC through case-control studies, a longitudinal study has provided an evidence that the genera *Moryella* and *Vibro* are specific microbes in early gastric neoplasia (EGN), suggesting that changes in the gut microbiota can be used as progressive biomarkers (52). Two metabolites from the gut microbiota were identified to distinguish GC tissues from non-GC tissues, with a high area under the curve (62).

## 7 Conclusions and future perspectives

In addition to *Hp*, many types of microbes are involved in the development, progression, prognosis, and treatment response of GC. However, no explicit microbiota and their metabolistes have been identified as a predominant indicator of GC development and ICI responses across published studies (121), and the mechanisms of microbial influence on GC are still unclear. Therefore, we summarized recent studies on bacteria and their metabolites that function mainly in GC progression and shed light on their significance in the diagnosis and treatment of GC. The mechanisms by which the microbiota modulate the progression of GC require further investigation. Furthermore, as the sequencing outcomes become more precise and the procedures cost lower, the dominant bacteria in GC tissues, gastric mucosa, and feces will be clarified. Large prospective cohort studies are needed to explore the mechanism of gut microbiota function in GC, as the intestinal flora changes dynamically during the long-term progression of GC.

The influence of the microbiota on immunotherapy has attracted increasing attention in the development of novel therapeutic approaches, such as FMT, probiotic supplement, and dietary immune-stimulating products. In this review, we summarize the potential regulatory function of the gut microbiome and metabolites on the immune response in GC, which indicates that favorable bacteria in patients with GC might increase systemic and antitumor immune responses. Moreover, FMT in immunotherapy responders may help reduce the side effects of ICIs. Therefore, it is important to explore microbial therapy combined with immunotherapy to improve the therapeutic effects and survival rate of GC in clinical settings.

## Author contributions

ZL and YX contributed to the conception and the main ideas of this study. MW drafted the manuscript, figures, and tables. GY, YT, and QZ helped write the article. ZL and YX reviewed and modified the manuscript. All authors contributed to the article and approved the submitted version.
